# Management of malignant breast phyllodes tumor with rib invasion during pregnancy: a rare case report and literature review

**DOI:** 10.3389/fonc.2025.1703191

**Published:** 2026-01-05

**Authors:** Sijie Yu, Panni Li, Wenwen Wu, Xianan Guo, Huihui Chen, Kexin Liu, Dan Ye, Zhihua Teng, Wei He, Liquan Wang, Yunxiang Zhou, Yiding Chen

**Affiliations:** 1 Nursing Department, The Second Affiliated Hospital of Zhejiang University School of Medicine, Hangzhou, Zhejiang, China; 2 Department of Breast Surgery and Oncology, The Second Affiliated Hospital, Zhejiang University School of Medicine, Hangzhou, Zhejiang, China; 3 Cancer Institute (Key Laboratory of Cancer Prevention and Intervsention, China National Ministry of Education), The Second Affiliated Hospital, Zhejiang University School of Medicine, Hangzhou, China; 4 Department of Thoracic Surgery, The Second Affiliated Hospital, Zhejiang University School of Medicine, Hangzhou, Zhejiang, China; 5 Department of Anesthesiology, The Second Affiliated Hospital, Zhejiang University School of Medicine, Hangzhou, Zhejiang, China; 6 Department of Obstetrics, The Second Affiliated Hospital, Zhejiang University School of Medicine, Hangzhou, Zhejiang, China

**Keywords:** malignant phyllodes tumor, pregnancy, breast cancer, surgery, multidisciplinary team

## Abstract

Phyllodes tumors of the breast are rare fibroepithelial neoplasms accounting for less than 1% of breast tumors, with malignant phyllodes tumors (MPTs) representing approximately one-quarter of cases. Occurrence during pregnancy is exceedingly uncommon and may be associated with accelerated tumor growth. We report a 32-year-old woman at 15 weeks of gestation with a two-year history of recurrent MPT who had undergone four prior excisions. During the current pregnancy, the tumor recurred with rapid progression and rib invasion. After multidisciplinary evaluation, radical extended resection with chest wall reconstruction using the latissimus dorsi muscle was performed in the second trimester without perioperative complications. The pregnancy continued uneventfully, and a healthy full-term infant was delivered. At six-month follow-up, no local recurrence or metastasis was detected. Herein, we present the first documented case of complete resection of a rib-infiltrating MPT during pregnancy and provide a comprehensive synthesis of the existing literature to inform the characteristics and comprehensive management of MPTs, with particular emphasis on surgical strategies. We also summarize available evidence on pregnancy-associated MPTs to characterize their clinical and biological features. These insights may inform individualized treatment planning and optimize the overall management of these rare tumors.

## 1 Introduction

Phyllode tumors of the breast (PTBs) are rare fibroepithelial neoplasms characterized by a distinctive leaf-like histological pattern (1). They account for 2.5% of all fibroepithelial lesions of the breast and 0.3% to 1% of all primary breast tumors (2, 3), with a higher prevalence in Asia (4, 5), most commonly occurring in women aged 35 to 55 years (6, 7). Although relatively uncommon, these tumors exhibit a high propensity for recurrence (8). PTBs typically present as rapidly growing, painless masses, averaging 3 to 5 cm in size. In cases of large tumors, conspicuous congested veins, skin surface ulceration, or chest wall invasion may be observed. According to the latest WHO classification criteria in 2019, PTBs are pathologically categorized into benign, borderline, and malignant based on stromal cellular atypia, the proportion of mitotic figures, the degree of stromal overgrowth and necrosis (9). Among these, malignant phyllodes tumors (MPT) constitute approximately 25% of cases, with local recurrence rates of 23% to 30% and a distant metastasis rate of around 9% (1, 2). Despite ongoing controversies regarding the comprehensive management of MPT, adequate surgical excision remains the standard approach to achieve optimal local control (10). This study reports a rare and highly challenging case of recurrent MPT complicated by rapid progression and rib invasion, necessitating complex surgical management aimed at achieving complete tumor resection while preserving gestation. Beyond the case illustration, we provide a literature review to delineate the clinicopathological characteristics and comprehensive management of MPTs, with a focus on surgical approaches and on the specific considerations for pregnancy-associated cases.

## 2 Case presentation

**Figure 1:**
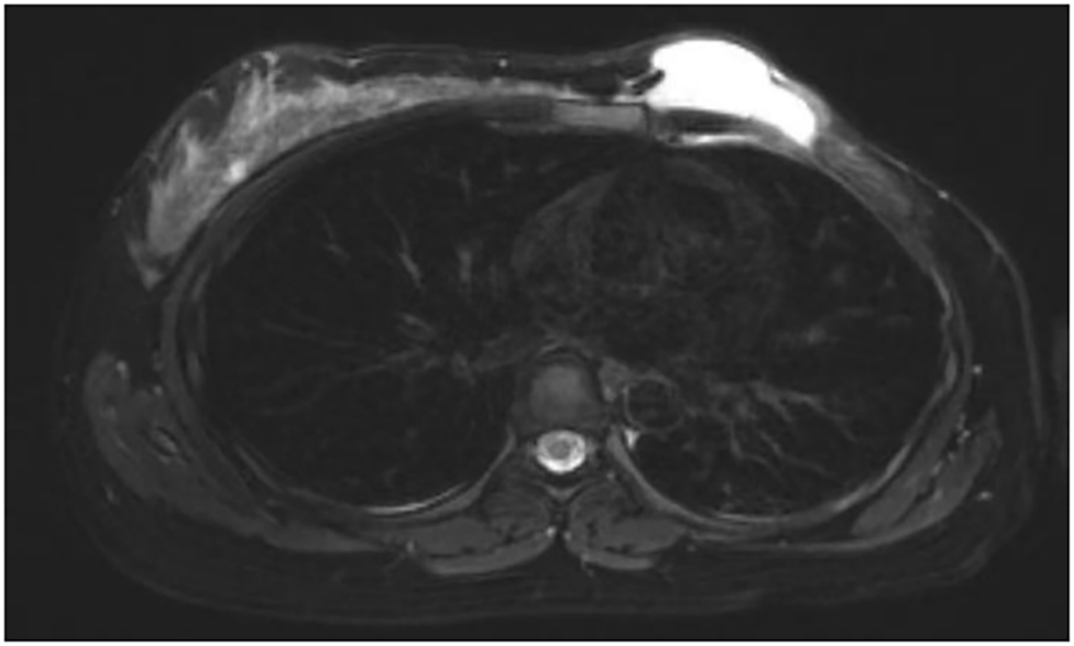
Preoperative breast MRI imaging. Transverse T2 revealed a left chest wall mass suggesting recurrence of a phyllode tumors of the breast, classified as BI-RADS 4C, with invasion into the chest wall and close proximity to the pleura.

A 32-year-old woman was admitted to the hospital with a chief complaint of enlargement and recurrence of a left breast mass for two years. Prior to this, she underwent multiple surgeries due to recurrent left breast masses, with specific diagnostic and treatment processes outlined in **Table 1**. It was two years ago when the patient first discovered a left breast mass during a routine physical examination. She underwent a segmental resection of the mass, with postoperative pathology revealing a benign PTB. One year later, during a follow-up visit, the left breast mass recurred. A biopsy confirmed the presence of an MPT, with no evidence of lymph node metastasis in the axilla. Consequently, the patient underwent a total mastectomy of the left breast, postoperative pathology indicated an MPT measuring approximately 2*1.5*1.0 cm. No adjuvant chemotherapy or radiotherapy was administered after the surgery. The patient became pregnant over two months ago, during which a nodule on the left chest wall was detected. She subsequently underwent two excisional surgeries under local anesthesia at an external hospital, with postoperative pathology confirming an infiltrative recurrence of the MPT. The patient is now experiencing skin ulceration and mass formation on the left chest wall and is currently 15 weeks and 4 days of gestation. She was admitted for further treatment of the left breast MPT as well as to preserve the pregnancy. As shown in **Figure 1**, preoperative chest magnetic resonance imaging (MRI) revealed a left chest wall mass suggesting recurrence of a PTB, classified as BI-RADS 4C, with invasion into the chest wall and close proximity to the pleura. Given the aggressive growth rate of this dangerous tumor, surgical excision to prevent further growth and metastasis is the primary objective.

**Table 1:** Disease development process and the treatment line.

Time	Disease development	Size (cm)	Surgical approach	Pathology	Ki-67
2021.7	Simple mass in the left breast	4*2	Simple lumpectomy of the left breast mass	Benign	NA
2022.11	Recurrence of a mass in the left breast	2*1.5*1	Left mastectomy	Malignant	30%+
2023.10.11	Left chest wall local recurrence	NA	Excision of the lesion in the left chest wall^1^	Malignant	50%+
2023.11.16	Left chest wall local recurrence	NA	Excision of the lesion in the left chest wall^1^	Malignant	80%+
2023.12.13	Recurrence on the left chest wall with invasion into the ribs	4*3.5	Radical extended surgery of the left chest wall	Malignant	60%+

^1^Local anesthesia.

NA, not available.

**Figure 2:**
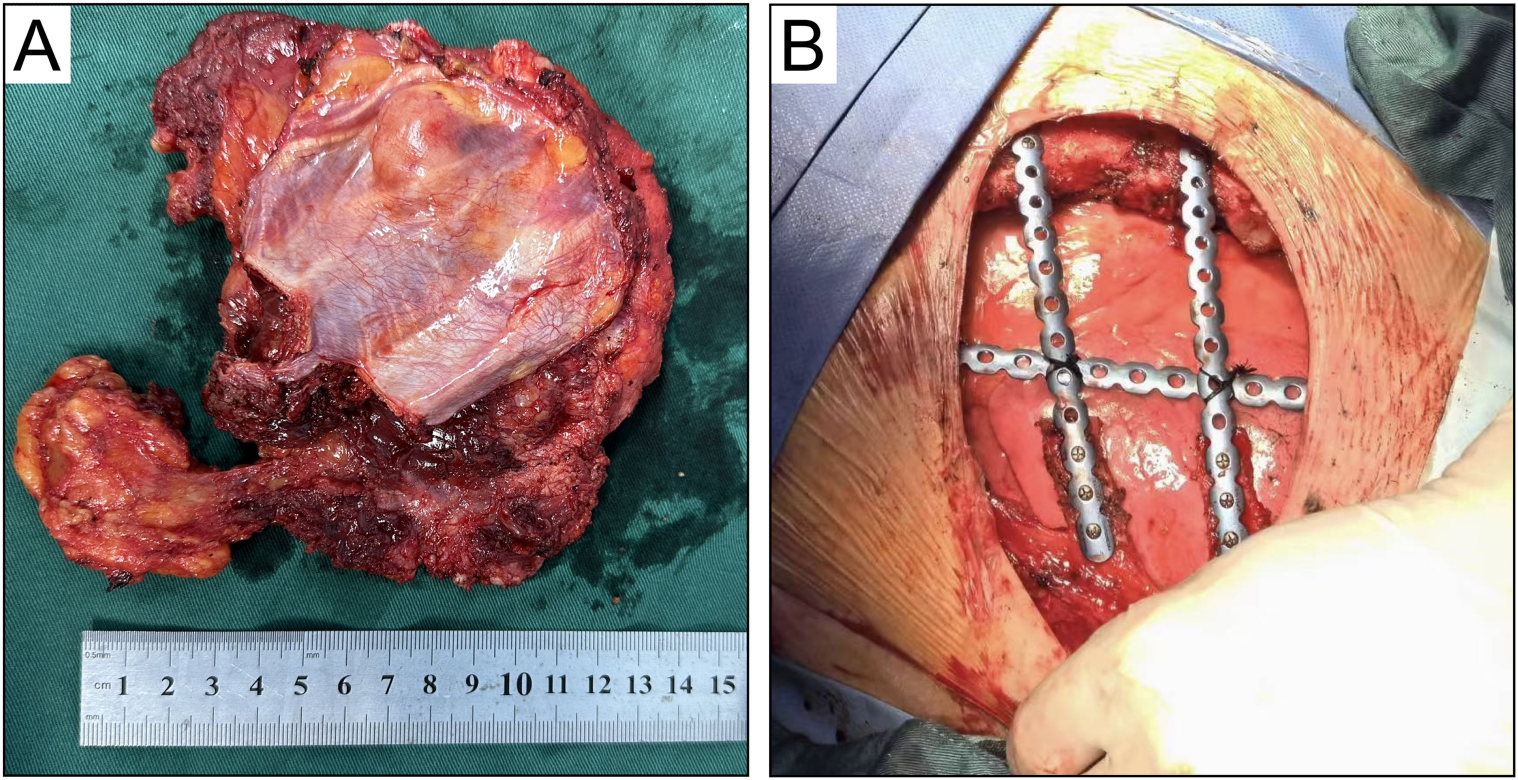
Radical extended resection of tumor and chest wall reconstruction. **(A)** specimen of left breast malignant phyllodes tumor after extended resection; **(B)** Removed the left 4–5 ribs and performed chest wall reconstruction with titanium plates.

**Figure 3:**
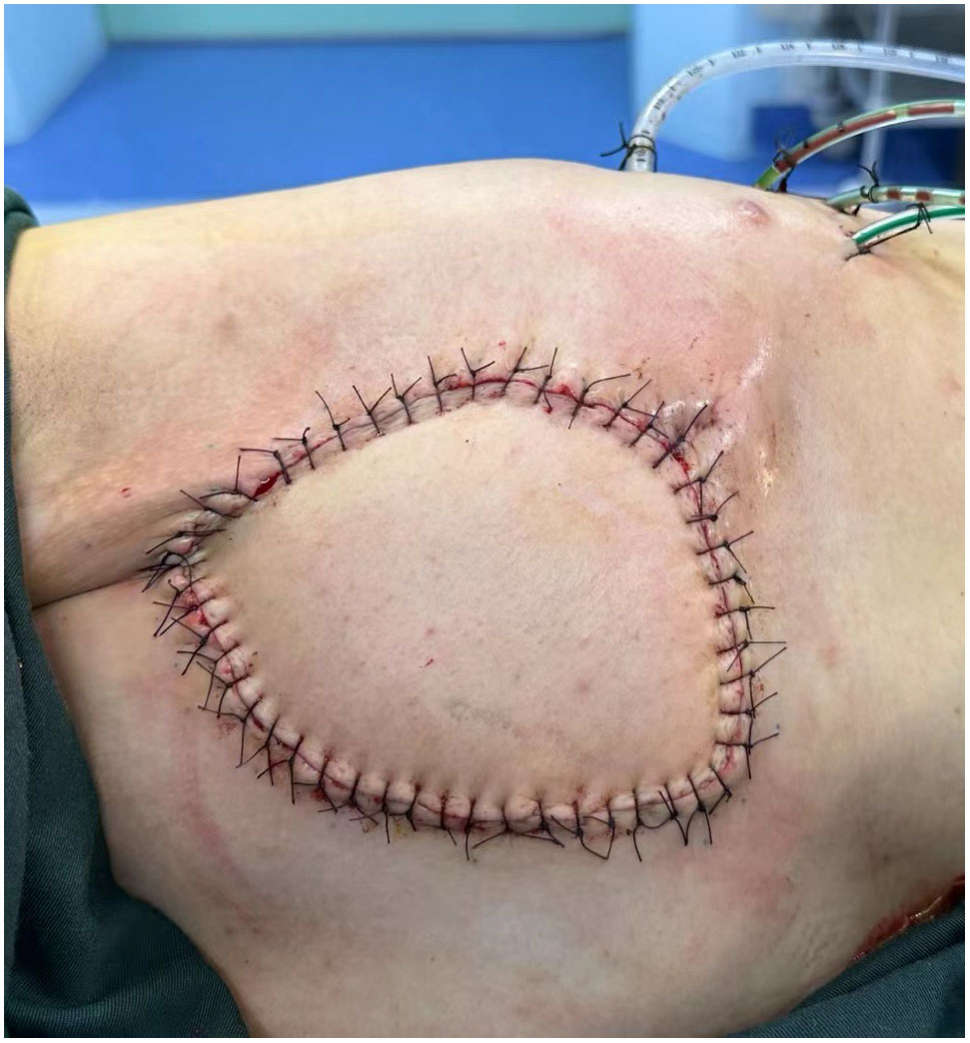
Early postoperative aspect after radical extended resection of the tumor, with left breast reconstruction using the latissimus dorsi muscle.

**Figure 4:**
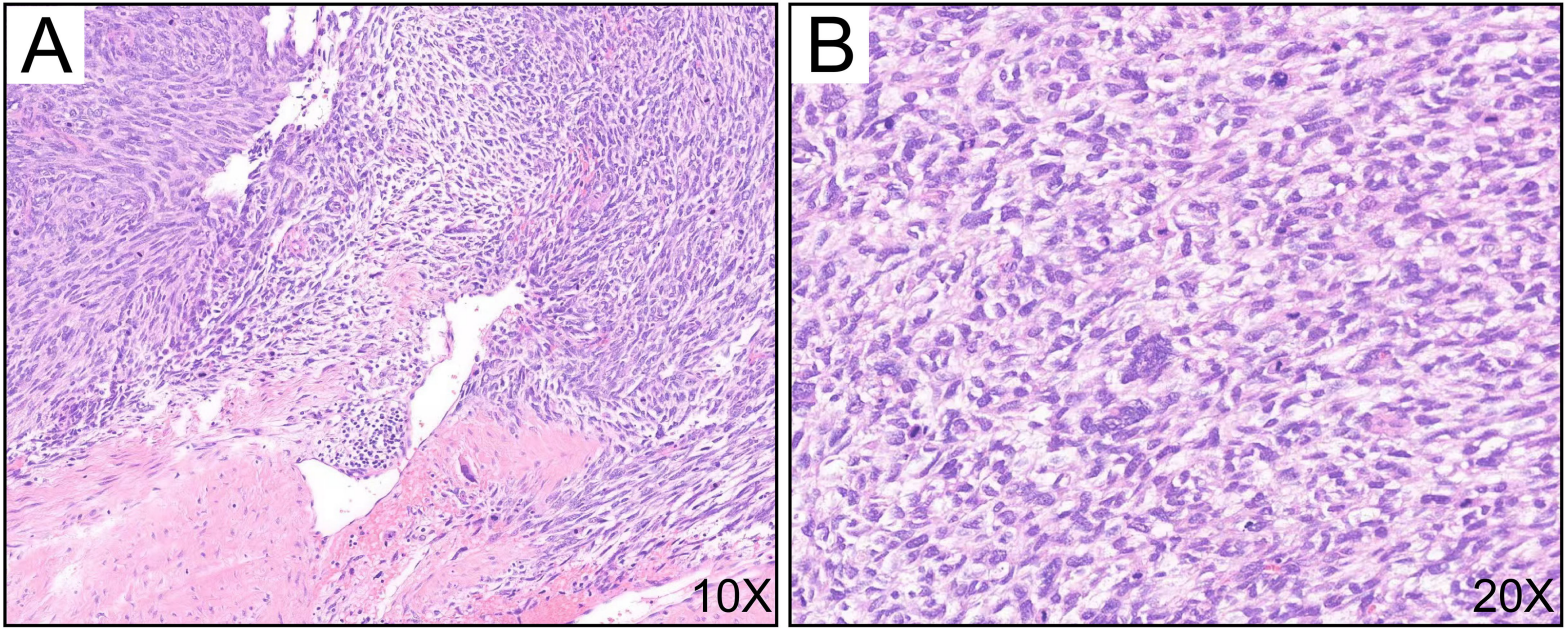
H and E staining of the tumor. Recurrence of breast malignant phyllodes tumor, demonstrating infiltrative tumor growth, with marked atypia of tumor cells and a markedly increased cellularity (>10/HPF). **(A)** H and E staining; magnification, ×10; **(B)** H and E staining; magnification, ×20.

Considering the patient’s mid-pregnancy status and the tumor’s invasion into the chest wall and ribs, the breast surgery team initiated a preoperative MDT discussion involving thoracic surgery, obstetrics, anesthesiology, and orthopedics. All disciplines conducted a comprehensive analysis of the treatment plan. MPTs are not sensitive to chemotherapy or radiotherapy (11), and given the patient’s pregnancy and elevated hormone levels that could promote rapid tumor growth, the surgical indication was clear, with the primary goal being radical tumor excision. Thus, with the support of various specialties, the patient underwent “radical excision of the left chest mass” under general anesthesia on December 13, 2023. The surgery lasted 4 hours, and through careful intraoperative monitoring and meticulous handling, the malignant tumor was completely excised (**Figure 2A**). The surgery team removed the left 4th and 5th ribs and performed chest wall reconstruction with titanium plates (**Figure 2B**). Then the latissimus dorsi muscle was utilized to fill the chest wall defect (**Figure 3**), and the fetus was not significantly affected during the perioperative period, continuing to grow well. Postoperative pathology confirmed a recurrence of MTB (**Figure 4**), with a 1 cm excision margin.

Postoperatively, the patient recovered well. She successfully delivered a healthy child on May 24, 2024. Follow-up outpatient visits over the next six months indicated good recovery, with no signs of local recurrence or distant metastasis detected.

## 3 Discussion and literature review

MPTs are rare and typically require aggressive surgical management due to their tendency for recurrence and metastasis. Adequate surgical excision with clear margins remains the standard approach for non-metastatic MPT (10). According to the National Comprehensive Cancer Network (NCCN) guidelines, the optimal approach for conservative surgery involves excising a margin of at least 1 cm or more (12). A negative margin serves as an independent prognostic factor for disease-free survival (DFS) and local recurrence in PTB. For malignant and borderline tumors, wider excision is recommended, as shown in the guideline, the risk of recurrence and metastasis is higher when margins are less than 1 cm. In this case, the patient had already undergone a total mastectomy during previous recurrences, and the current lesion had infiltrated the ribs from the residual chest wall. To achieve complete excision and minimize the risk of recurrence, a radical resection involving the chest wall and the 4th and 5th ribs was performed, with both superior and inferior margins being negative. In cases of significant tissue defects following large PTB excision, reconstructive techniques such as tissue flap transfer can be employed, as demonstrated by the latissimus dorsi flap reconstruction in this case. Previous studies indicate that the axillary involvement rate of MPT is only 1% to 3% (3, 13, 14). As the therapeutic value of axillary lymph node (ALN) removal remains uncertain, ALN dissection or surgical staging of the axilla is generally not performed unless clear clinical or pathological evidence of ALN involvement exists (10). Given that preoperative imaging in this patient indicated no significantly enlarged or abnormal ALNs, we did not perform ALN biopsy or dissection.

While the established role of surgical excision in treating MPT is undeniable, the significance of postoperative radiotherapy continues to be uncertain. Two large retrospective studies, in which 455 of 2261 and 458 of 3210 patients received adjuvant radiotherapy, respectively, reported no improvement in long-term survival for MPT (15, 16), although adjuvant radiotherapy was associated with a significant reduction in local recurrence (16, 17). Similarly, a meta-analysis highlighted that while adjuvant radiotherapy effectively improves local disease control in PTB, it does not confer a survival benefit (18). Currently, adjuvant radiotherapy may be selectively considered for high-risk MPT, such as tumors with high-grade histology, size >5 cm, or positive surgical margins (3). Furthermore, the use of adjuvant chemotherapy remains controversial due to its limited clinical benefits (19). However, when the tumor metastasizes or surgery is not feasible, chemotherapy and radiotherapy appear to reduce recurrence rates and prevent disease progression. In this case, the patient did not undergo adjuvant radiotherapy or chemotherapy postoperatively and remained in good health during follow-up.

Beyond being a rare instance of PTB, our case is particularly distinctive due to the involvement of a rapidly growing MPT occurring in the first trimester of pregnancy. Pregnancy-associated breast cancer (PABC) is defined as breast cancer diagnosed during pregnancy or within one year postpartum (20). In cases of early-stage PABC, if postoperative treatment such as radiotherapy or chemotherapy is not required, the pregnancy may continue. For PABC, there is no evidence suggesting that therapeutic abortion improves prognosis (21). Treatment plans should be individualized, and prior to starting therapy, each pregnant patient should be thoroughly informed about the treatment options, their impact on pregnancy, and potential teratogenic effects. The decision to continue or terminate the pregnancy is a personal one. Surgical management of breast cancer during pregnancy should follow the same guidelines as for non-pregnant women. However, due to the increased risk of miscarriage, it is generally recommended to postpone surgery until after the first trimester (22). As mentioned above, MPT can exempt patients from chemotherapy and radiotherapy, so despite the lack of formal guidelines, continuation of pregnancy with MPT can still be considered. Specifically, the patient in this case, who had not conceived in six years of marriage and had previously undergone curettage for a missed abortion, was determined to preserve the pregnancy after being informed of the associated risks. Given the necessity for surgical intervention and the imperative to ensure maternal-fetal safety, an MDT approach was employed. Ultimately, with the cooperation of various specialties, the surgery was successfully completed. The MDT model allowed for optimal allocation of healthcare resources, enhanced quality control, and facilitated interdisciplinary decision-making, ultimately improving the patient’s clinical outcome.

Actually, PTB during pregnancy accounts for only 25% of all PTBs (23), while MPT is even rarer. A comprehensive review pertaining to pregnancy-associated breast MPT was conducted of the existing literature. Overall, fewer than 20 case reports of MPT during pregnancy and lactation have been reported, but there are some interesting consistencies. Information from each case report is summarized in **Table 2** (24–41). The mean age of the patients was 31.6 years, with 50% diagnosed during the first or second trimester of pregnancy. The average size of these malignant tumors is 12.8 cm (ranging from 1.5 to 32cm), with most exhibiting a rapid growth rate during pregnancy. Notably, 17.8% of the cases were recurrent tumors. Similarly, the patient in this case experienced multiple recurrences of the PTB. Despite four local excisions, the tumor continued to infiltrate the ribs. Prognostic factors influencing disease recurrence include tumor size, grade, postmenopausal status, necrosis, cellular atypia, infiltrative margins, and positive surgical margins (42). In these reports, it is suspected that pregnancy played a significant role in the recurrence. Hormonal changes during pregnancy may stimulate proliferative changes in breast glandular tissue, such as lobular and acinar growth, potentially contributing to both recurrence and accelerated tumor progression. As shown in **Table 2**, 14 out of 18 MPTs (77.8%) exhibited rapid enlargement during pregnancy or lactation, highlighting the potential hormone dependence of MPTs. However, due to the relatively limited number of MPTs during pregnancy, further research is needed to confirm a definitive link between pregnancy-related hormonal changes and MPT pathogenesis. Moreover, due to the characteristic rapid growth of MPT during pregnancy, breast examination in early pregnancy is particularly important. Among previously reported cases, only three patients underwent phyllodes tumor resection in the mid-early stages of pregnancy (25, 26, 38), with the earliest case involving a partial mastectomy at 11 weeks of gestation (25). Early detection and timely management theoretically increase the rate of breast conservation and reduce the occurrence of complications.

**Table 2:** Case reports of malignant phyllodes tumor occurring during pregnancy and lactation.

First author, year	Age, year	Gestation presentation	Size (cm)	Recurrence	Rapid growth in pregnancy	Operation period	Surgical approach	Pathology	Follow up
Bal 2012 (24)	32	Postpartum period	Whole breast	Y	Y	NR	SA+SLNB+IBR	Malignant	6 m: free of disease
Blaker 2010 (25)	27	9th week	4	N	N	11 weeks gestation	WLE	Malignant	8 m: free of disease
Hernanz 2018 (26)	35	14th week	7.6 (R) 1.7 (L)	N	Y	14 (R)/23 (L) weeks gestation	RB: SA+IBR LB: SA+IBR	Malignant	Safe before labor^1^
Kelten 2016 (27)	37	4 weeks after labor	13	N	Y	4 weeks after labor	SM	Malignant	NR
Lee 2018 (28)	21	NR	2.9	N	N	NR	Lumpectomy	Malignant	NR
Li 2008 (29)	29	6 months after labor	Whole breast (36*28)	Y	Y	6 months after labor	SM; then WLE and flap	Malignant	NR
Mrad 2000 (30)	32	NR	9 (R), 21 (L)	N	NR	NR	RB: WLE LB: SM	RB: Benign LB: malignant	17 m: free of disease
Mustață 2021 (31)	36	19th week	10	N	Y	35 weeks gestation	SM	malignant	4 y: free of disease
Narla 2018 (32)	28	Postpartum period	14	N	Y	NR	lumpectomy; then SM	Malignant	NR
Nejc 2008 (33)	28	34th week	18	N	Y	2 weeks after labor	WLE	Malignant	20 m: free of disease
Pacchiarotti 2011 (34)	41	17th week	6	N	Y	During pregnancy	WLE	Malignant	NR
Pasta 2012 (35)	43	T2	1.5	N	N	During pregnancy	WLE	Malignant	1 y: free of disease
Pytel 2009 (36)	25	T2	NR	N	Y	2 years after labor	SM	Malignant	NR
Ray 2011 (37)	24	36th week	22	N	Y	8 weeks after labor	SM	Malignant	NR
Simpson 2007 (38)	29	20th week	17	N	Y	22 weeks gestation	WLE+SLNB	Malignant	NR
Tortoriello 2017 (39)	37	T1	24	N	Y	NR	Lumpectomy	Malignant	2.5 y: free of disease
Vergine 2012 (40)	27	Postpartum period	10	N	Y	NR	SM	Malignant	1 y: free of disease
Zhang 2021 (41)	38	T1	6	Y	Y	3 years after labor	SM	Malignant	1.5 y after surgery, lung metastasis

^1^During pregnancy, the patient did not have any complications.

NR, not reported; T1, 1-12weeks; T2, 13–27 weeks; T3, 28–40 weeks; Y, yes; N, no; RB, right breast; LB, left breast; SM, simple mastectomy; WLE, wide local excision; SA+IBR, subcutaneous adenectomy and immediate breast reconstruction; SA+SLNB+IBR, subcutaneous adenectomy, sentinel lymph node biopsy and immediate breast reconstruction.

Notably, PTB can also metastasize, posing significant challenges in treatment. PTB primarily metastasizes through hematogenous rather than lymphatic routes (43), with metastases mainly observed in patients with MPT. The most common metastatic sites for MPT are the lungs and bones, though metastases to adrenal glands and the brain can occur concurrently (44). Once patients with MPT develop distant metastases, the prognosis is extremely poor, with a median survival time of 10.7-11.5 months (43). Therefore, gaining a deeper understanding of the mechanisms underlying the development and progression of PTB is crucial. Dios et al. (45) proposed that PTBs may be associated with MED12 mutations. Additionally, there are theories suggesting that tumor-associated macrophages promote the transformation of PTB into malignant forms by facilitating fibroblast activation (46). For individuals with recurrent disease or high-risk factors, relevant genetic testing can be conducted to explore potential molecular biological therapeutic targets for PTB. Furthermore, patients with MPT during pregnancy are typically younger, necessitating the consideration of hereditary breast cancer risks. Genetic testing may also guide patients in managing reproductive choices and assessing hereditary breast cancer risks for future generations.

This study has inherent limitations. As a case-based review, this study is restricted by the single-patient nature of the report, which limits the generalizability of its observations, particularly in complex clinical settings such as malignant phyllodes tumors occurring during pregnancy. Robust evidence on this topic remains scarce, and higher-level studies including prospective multicenter cohorts, randomized controlled trials when feasible, and meta-analyses based on such data are needed to refine clinical guidelines and clarify optimal management strategies. Until stronger evidence becomes available, treatment decisions should be individualized with careful consideration of tumor biology, expected surgical outcomes, maternal and fetal factors, and patient preferences.

## 4 Conclusions

In summary, this study reports a rare case involving a patient with an MPT of the breast that recurred and affected the ribs, while also pregnant at the time of admission. The patient underwent a complex radical resection while simultaneously requiring treatment to preserve the pregnancy. PTBs that develop during pregnancy typically exhibit a faster growth rate and may be prone to multiple recurrences or malignant transformation. In patients with rapidly growing breast tumors, particularly during pregnancy, there should be a high suspicion for MPT. Multiple recurrences can alter the nature and invasiveness of PTBs, making vigilance crucial when signs of recurrence appear. This is especially important for women of childbearing age or those planning pregnancy, who should undergo breast examinations prior to ruling out any potential risks, enabling early detection and timely, effective treatment. To reduce the risk of tumor recurrence, standardized treatment strategies need to be established. Complete surgical excision remains the preferred treatment for non-metastatic PTB, while the efficacy of adjuvant radiotherapy and chemotherapy in PTB management remains uncertain. In complex cases, such as those involving recurrent breast tumors during pregnancy, an MDT approach is particularly crucial for standardized diagnosis and treatment. Future clinical studies with more in-depth analysis are needed to explore the diagnosis and treatment of PTB at various stages.

## Data Availability

The original contributions presented in the study are included in the article/supplementary material. Further inquiries can be directed to the corresponding authors.
